# Incidence of Gastroesophageal Reflux in Dogs Undergoing Orthopaedic Surgery or Endoscopic Evaluation of the Upper Gastrointestinal Tract

**DOI:** 10.3390/vetsci7040144

**Published:** 2020-09-25

**Authors:** Carlotta Lambertini, Marco Pietra, Giorgia Galiazzo, Francesco Torresan, Stefania Pinna, Luciano Pisoni, Noemi Romagnoli

**Affiliations:** 1 Department of Veterinary Medical Sciences, University of Bologna, Italy. Via Tolara di Sopra 50, 40064 Ozzano dell’Emilia (BO), Italy; marco.pietra@unibo.it (M.P.); giorgia.galiazzo2@unibo.it (G.G.); stefania.pinna@unibo.it (S.P.); luciano.pisoni@unibo.it (L.P.); noemi.romagnoli@unibo.it (N.R.); 2 Department of Digestive System, St. Orsola-Malpighi Hospital, Via Massarenti, 40100 Bologna, Italy; francesco.torresan@aosp.bo.it

**Keywords:** anaesthesia, canine, endoscopy, gastroesophageal reflux

## Abstract

Gastroesophageal reflux (GER) is a common event during general anaesthesia but is often underdiagnosed in veterinary medicine. The oesophageal pH in anaesthetised dogs undergoing endoscopic evaluation of the upper gastrointestinal tract (END group; *n* = 12) or orthopaedic surgery (ORT group; *n* = 12) was measured using an oesophageal probe. The dogs were sedated with acepromazine or with methadone or butorphanol, and anaesthesia was induced with propofol and maintained with isoflurane. Of the 24 dogs in this study, 21 (87.5%) had an episode of GER during anaesthesia. The incidence of GER, as well as the first, the minimum, and the maximum pH values, did not differ significantly between the groups. The mean maximum difference versus the first pH value was higher for dogs in the END group (−2.6 ± 3.5) as compared with those in the ORT group (−0.7 ± 2.5), although they were not statistically significant (*p* = 0.25). The administration of methadone or butorphanol had no significant effect on the development of acidic reflux or biliary reflux. In the acepromazine-sedated dogs, the incidence of GER did not differ significantly between patients undergoing an endoscopic procedure and those undergoing orthopaedic surgery; however, during endoscopy, fluctuations in the oesophageal pH can be expected, even without any clinical signs of GER.

## 1. Introduction

Reflux of gastric content into the oesophagus is a common event in humans and in veterinary patients undergoing general anaesthesia. This condition, defined as gastroesophageal reflux (GER), occurs in 13.3 to 60% of dogs under general anaesthesia [1,2,3,4].

Gastroesophageal reflux is underdiagnosed in veterinary medicine; when occurring in the peri-anaesthetic period, GER is not usually apparent and is not related to vomiting [1,2,3]. However, post-anaesthetic esophagitis, oesophageal strictures, and aspiration pneumonia, the most common GER-associated complications, are characterised by high morbidity, and mortality up to 23% [3,5,6,7]. In humans, as in veterinary medicine, the measurement of oesophageal pH, close to the gastroesophageal sphincter, is a commonly used method for the diagnosis of GER [8].

The pathophysiologic mechanism causing anaesthesia-related GER is still under debate; however, several predisposing factors have been correlated with this condition in anaesthetised dogs: the duration of the preoperative fasting time and the type of food administered [1,9], late pregnancy [10], recumbency of the animal during anaesthesia [3] and the anaesthetic protocol used [1,3]. On the contrary, breed, sex, and age do not seem to significantly influence the incidence of GER [2]. The effects of the type of the procedure on the incidence of GER has also been investigated. In detail, reflux occurred more frequently in dogs undergoing intra-abdominal surgery as compared with those undergoing a non-abdominal procedure [2].

Endoscopic examination of the gastrointestinal tract is a procedure often performed in dogs for diagnostic purposes [11]. Endoscopic examination of the gastrointestinal tract for diagnosing canine inflammatory bowel disease (IBD) includes both an upper gastrointestinal examination (oesophagus, duodenum, and stomach) and a lower intestinal examination (colon, ileum, and caecum) [12,13]. During the upper gastrointestinal endoscopy procedure, conditions capable of inducing gastric or duodenal reflux into the oesophagus could depend directly on the distension of the lower oesophageal and pyloric sphincters during passage of the endoscope, as well as indirectly on the increase of the pressure inside the bowel due to the introduction of gas or water to widen the stomach and the intestinal loops [14].

Reflux of the gastrointestinal content is an expected complication after endoscopy [15]; however, clinical studies regarding this topic are lacking.

To the authors’ knowledge, there are no data evaluating the oesophageal pH of the lower oesophagus in dogs undergoing gastro-duodenoscopy.

The aim of the present study was to detect the oesophageal pH of the lower oesophagus and to evaluate the incidence of GER in dogs undergoing endoscopic procedures of the upper gastrointestinal tract. A second aim of the study was to compare these results with those obtained in dogs undergoing non-abdominal procedures and, therefore, dogs undergoing orthopaedic surgery of the hind limbs were taken into consideration. The authors hypothesised that the incidence of GER would be higher in dogs undergoing endoscopy.

## 2. Materials and Methods

### 2.1. Animals

Twenty-four dogs admitted to the Veterinary Teaching Hospital of the University of Bologna for endoscopic procedures of the upper gastrointestinal tract (END group; *n* = 12) or elective orthopaedic surgery on the hind limbs (ORT group; *n* = 12) were included in the study. The exclusion criteria were dogs weighing less than 10 kg, dogs with vomiting and regurgitation in the 24 h prior to the procedure, and dogs with a diagnosis of a hiatal hernia (types II or III).

The study was conceived as a clinical trial in which dogs requiring anaesthesia for scheduled orthopaedic or endoscopic procedures were included. The placement of the probe for measuring the pH did not produce any suffering to the animals. The study was performed in accordance with the disposition of the University Ethics Committee of the University of Bologna, conforming with the D.lgs 26/2014 of the Italian government.

### 2.2. Anaesthetic Protocol

Food was withheld for 12 hours before premedication while water was available ad libitum. All the dogs were premedicated with acepromazine (Prequillan; ATI, Bologna, Italy) 0.02 mg/kg intramuscularly (IM) in combination with methadone (Semfortan; Dechra, Turin, Italy) 0.3 mg/kg or butorphanol (Nargesic; Acme S.r.l., Reggio Emilia, Italy) 0.2 mg/kg. Methadone or butorphanol were administered at the anaesthetists’ preference. Fifteen minutes after premedication, we placed an intravenous catheter into the cephalic vein. General anaesthesia was induced with propofol (Proposure; Boheringer Ingelheim, Milan, Italy), administered intravenously (IV) and titrated to effect in order to achieve endotracheal intubation. General anaesthesia was maintained with isoflurane in 100% oxygen delivered through a rebreathing system.

In the dogs undergoing orthopaedic surgery, we provided intraoperative analgesia with epidural anaesthesia combining 2% lidocaine (Lidocaina cloridrato; S.A.L.F., Bergamo, Italy) (4.4 mg/kg) and 0.5% ropivacaine (Naropina; AstraZeneca, Milano, Italy) (2.2 mg/kg) up to a total volume of 0.25 mL/kg. The epidural injection was administered between the L7-S1 vertebrae using a spinal needle after sterile preparation of the field. The correct placement of the spinal needle was evaluated using the loss of resistance technique. Both intubation and epidural puncture were performed, maintaining dogs in sternal recumbency.

### 2.3. Anaesthetic Monitoring

After induction, the monitoring devices from a multiparametric monitor were attached (Datex-Ohmeda- S3; Datex-Ohmeda Inc., Madison, WI, USA). The cardiorespiratory parameters were monitored and recorded every 5 minutes. All the dogs were evaluated during the procedures to detect signs of pain. An increase in heart rate (HR) or in mean arterial pressure (MAP) values greater than 20% as compared with the values recorded before the application of the stimulus were considered indicative of inadequate analgesia. In this case, an additional bolus of methadone (0.1 mg/kg; Semfortan; Dechra Veterinary products srl, Torino, Italy) or butorphanol (0.1 mg/kg; Alvegesic; Dechra Veterinary products srl, Torino, Italy) was administered to the dogs in the END group while ketamine 0.6 mg/kg/h was administered to the dogs in the ORT group. In addition, buprenorphine (Buprenodale; Dechra Veterinary products srl, Torino, Italy) (0.015 mg/kg IM) and carprofen (Rimadyl; Zoetis Italia srl, Roma, Italy) (4 mg/kg subcutaneously) were administered at the completion of the surgery soon after extubation. Dogs requiring additional rescue intraoperative analgesia were excluded from the study. At the end of the procedures, we monitored the recovery from the anaesthesia until they were fully awake.

### 2.4. Oesophageal pH Measurement

A disposable flexible oesophageal probe was used for oesophageal pH monitoring (pH day 2-Menfis; Biomedica, Bologna, Italy). The probe was calibrated 1 hour prior to the procedure, using buffer solution at standard pH values (pH 7.0 and pH 4.0). Soon after intubation, we introduced the probe into the oesophagus for a length equal to the distance between the incisor tooth and the cranial border of the 10th rib, as previously described [3,16]. The probe was then fixed to the canine tooth with tape in order to prevent its dislodgement. The probe was removed at the end of the endoscopy of the upper gastrointestinal tract in the END group or immediately before extubation in the ORT group. The correct positioning of the probe was confirmed directly under endoscopic view in the END group dogs, or by fluorospic evaluation (lateral view of the thorax) in the ORT group dogs. Fluoroscopic examination was carried out, passing through the surgical table from below, before the preparation of the surgical field. After placement, the probe was connected to a portable data logger, and pH measurement was continuously recorded every 1 s from the probe insertion up to its removal. The data were stored on a computer and analysed at the end of the study using computer software (PH/HS software, Medica SpA, Modena, Italy).

Oesophageal reflux was defined as a decrease in oesophageal pH to a value <4.0 (acidic reflux) or as an increase to a value >7.5 (biliary reflux) for at least 30 s, as previously described by Wilson and colleagues [3].

The following parameters of the oesophageal pH measurement were considered: the first pH value (the first value recorded after correct probe placement), the minimum pH value, the maximum pH value, and the maximum difference versus the first pH value.

All the dogs in the END group were maintained in left lateral recumbency. When the fluoroscopic examination was completed, the dogs in the ORT group were placed into the recumbency required for the surgical procedure. The recumbency of each dog was recorded on the anaesthetic record. The surgical table was always maintained parallel with the floor.

### 2.5. Statistical Analysis

The statistical analysis was carried out using MedCalc 6.3 computer software (MedCalc Software, Ostend, Belgium). The age and the weight of the dogs, the dosage of propofol used with respect to body weight, the length of the procedure, and pH measurement were evaluated for normality using the Shapiro–Wilk test. The normally distributed data were compared between groups using an independent samples *t*-test; not normally distributed data were compared using a Mann–Whitney test for independent samples.

The analysis of the pH values was carried out after calculating the average of the oesophageal pH values recorded every 5 minutes. Comparisons of the pH values were carried out between the groups by means of a *t*-test. Fisher’s exact test was used to test the differences in the categorical variables (sex and recumbency position) between groups. The calculation of the incidence of GER in the two groups was based on the presence or absence of acidic or biliary reflux in each dog, and Fisher’s exact test was used for comparison between the two groups. The effect of opioids on the incidence of GER was evaluated by defining an odds ratio (OR).

A *p* value < 0.05 was considered statistically significant.

## 3. Results

### 3.1. Animals and Procedures

All 24 dogs completed the study. A total of 37.5% of the dogs enrolled in the study were mixed breed dogs. The most represented pure breeds were boxers (two dogs), Dalmatians (two dogs), German shepherds (two dogs), and Rottweilers (two dogs) (Table 1). The distribution of the dogs’ breed did not differ significantly between the groups. There was no significant difference in age, weight, and sex between the groups. All the dogs in the END group had undergone gastroenteric endoscopy as part of the procedures for diagnosing IBD. All the dogs in the ORT group had a cranial cruciate ligament rupture and had undergone either the tibial tuberosity advancement or the tightrope technique.

All the dogs undergoing endoscopic evaluation were positioned in left lateral recumbency. The surgical procedures in the ORT group were performed by positioning the dogs in lateral recumbency (six in left lateral recumbency and four in right lateral recumbency) or in dorsal recumbency (2 out of 12). The surgical table was always maintained straight and parallel with the floor.

The anaesthesia time for gastroduodenal endoscopy was significantly shorter in the dogs in the END group (31.1 ± 9.9 min) than for those undergoing orthopaedic surgery (133.3 ± 34.6 min) (Table 1), *p* = 0 < 0.001.

The dosage of propofol administered for the induction of the general anaesthesia did not differ significantly between the two groups (Table 1), *p* = 0.58. None of the dogs required adjunctive rescue analgesia.

### 3.2. Oesophageal pH Measurement

**Figure 1:**
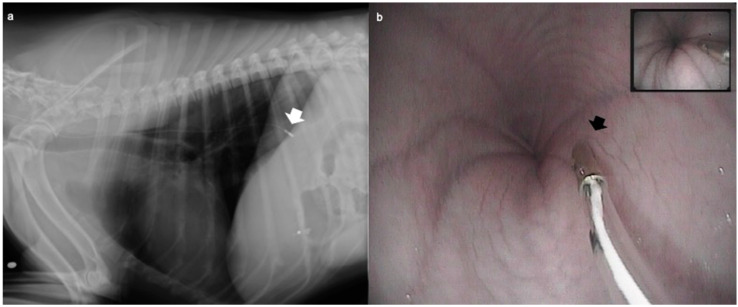
The correct placement of the oesophageal probe was confirmed by (**a**) fluoroscopy in the dogs undergoing orthopaedic surgery (ORT group) or (**b**) by endoscopy in the dogs undergoing an endoscopic examination (*n* = 12) (END group). After intubation, the probe was placed in the oesophagus up to a length previously measured externally and determined to be equal to the distance between the incisor tooth and the cranial border of the 10th rib.

The pH measurement probe was easily introduced in all patients within 5 min from intubation; the probe was correctly placed in all dogs as confirmed by endoscopic or fluoroscopic evaluation (Figure 1).

Data concerning oesophageal pH monitoring are reported in Table 2. Of the 24 dogs in this study, 21 (87.5%) had an episode of GER during anaesthesia (11 dogs in the END group and 10 dogs in the ORT group). In the END group, 75% of the dogs (9/12) experienced biliary reflux and 33.3% (4/12) experienced acidic reflux. In the ORT group, 50% (6/12) of the dogs experienced biliary reflux and 33.3% (4/12) experienced acidic reflux. Three dogs experienced both acidic and biliary reflux in the END group. The overall incidence of biliary or acidic reflux did not differ significantly between the two groups (*p* = 0.67 and *p* = 0.68, respectively).

No significant difference at the first pH value was noted between the two groups (*p* = 0.23); it was 8.2 ± 1.5 in the END group and 6.8 ± 2.1 in the ORT group. In the END group, the minimum pH value was 5.3 ± 3.3 and the maximum pH value was 8.6 ± 1.4. In the ORT group, the minimum pH value was 5.3 ± 2.8 and the maximum value was 7.5 ± 2.5. The minimum and the maximum pH values did not differ significantly between the two groups (*p* = 0.6 and 0.23, respectively).

The mean maximum difference versus the first pH value was higher for the dogs in the END group (−2.6 ± 3.5) as compared with those of the ORT group (−0.7 ± 2.5); however, the difference was not statistically significant (*p* = 0.25).

Fifty percent of the dogs in each group received methadone and the other 50% received butorphanol. The development of acidic reflux was not correlated with the administration of either methadone or butorphanol. However, the administration of methadone carried an increased risk of developing biliary reflux (OR 2.1; 95% CI 0.4 to 12.9; *p* = 0.4) but butorphanol did not (OR 0.5; 95% CI 0.08 to 2.7; *p* = 0.4); however, the correlation was not statistically significant.

Recovery was uneventful in all dogs. None of the dogs exhibited salivation, vomiting, or aspiration pneumonia at recovery. None of the dog owners reported signs related to esophagitis at 1 month after surgery.

## 4. Discussion

In the present study, the incidence of GER in dogs undergoing upper gastrointestinal endoscopy was evaluated for the first time, and the results were compared with those obtained in dogs undergoing a non-abdominal surgical procedure.

Although the oesophageal pH value during a gastroenteroscopy has never been evaluated in dogs, the results from previous studies involving dogs during surgery have suggested that the incidence of GER is higher in dogs undergoing abdominal procedures as compared with those undergoing non-abdominal procedures [2]. The authors hypothesised that intra-abdominal manipulations determined increased gastric pressure, which likely resulted in GER [2]. Similarly, during gastroduodenal endoscopy, gas insufflation, useful for the distension of the organs and the visualisation of the mucosa, can induce a marked pressure increase in the stomach and in the bowel [17]. An intragastric pressure exceeding 10 cm H_2_O has been defined as a risk for the development of GER [18,19]. Even if, under normal conditions, the increase in gastric pressure within normal limits is associated with an increased reflex in the lower oesophageal sphincter (LES) tone, this mechanism may be disrupted by several factors [19]. First of all, during endoscopy, the passage of the endoscope through the LES and the pyloric sphincter may lead to a leak of gastric or intestinal content up to the oesophagus. In the present study, the incidence of GER in dogs undergoing upper gastrointestinal endoscopy or orthopaedic surgery did not differ significantly; in fact, the majority of the dogs in the two groups had an episode of GER without statistically significant differences in the minimum pH value, the maximum pH value, and the difference between the last and the first pH values. However, despite the lack of a statistically significant difference between the two groups, the results indicated that the endoscopic procedures were associated with a greater variability in the pH value as compared with the orthopaedic procedures, as expressed by the maximum difference versus first pH value recorded. When a post hoc power analysis was carried out, it indicated a power of 33.4% in detecting a difference in the maximum difference versus the first pH value between the two groups. Therefore, it cannot be excluded that including more animals in the present cohort would have led to significant results.

Another factor affecting the incidence of reflux is fasting time [1]. In fact, the incidence of GER increased significantly with an increase in fasting time in dogs that fasted from 12 to 18 h up to 24 h, being at higher risk of developing reflux as compared with those which fasted for 2 to 4 hours [1]. In the present study, the dogs fasted for 12 h but the type of food previously administered was not taken into consideration; a previous study demonstrated that the type of food administered significantly influenced the gastric pH value [9].

Several authors have also investigated the effects of the anaesthetic protocols on the incidence of GER. In fact, anaesthetic drugs potentially interfere with the regulation of the sphincter tone [20,21,22]. In dogs, acepromazine administered at high doses (0.1 mg/kg) reduces the LES pressure up to 38.8% as compared with pre-treatment values [20,21]. In addition, opioid receptors (mu, kappa, and delta) are widely distributed in the enteric nervous system and have been identified in the LES and in the pyloric sphincter, even if with a different distribution between these two structures [23]. Therefore, opioids which are administered exogenously, can interfere with several gastrointestinal functions and with the regulation of the LES or the pyloric sphincter tone, among others [22,24]. In conscious human volunteers, morphine administered alone inhibited the relaxation of the LES [25]. However, in a population of dogs undergoing orthopaedic surgery, the authors observed that the addition of morphine (0.22 or 1.1 mg/kg) to acepromazine (0.044 mg/kg) increased the incidence of GER (50% and 60%, respectively) when compared with acepromazine administered alone (incidence of GER of 27%) [3]. A limitation of the present study was that no attempt was made to standardise the anaesthetic protocol used; all the dogs received acepromazine, and methadone or butorphanol were administered at the anaesthetists’ discretion. However, the administration of butorphanol or methadone was uniform in the two groups. The results indicated that only methadone administration was associated with an increased risk of developing biliary reflux, but the correlation was not statistically significant. This is in contrast with the results of a recent study in which methadone and butorphanol were compared as premedicant drugs for dogs undergoing endoscopic evaluations of the upper gastrointestinal tract [26]. In that study, McFadzean and colleagues observed that the incidence of oesophageal and especially of duodenal reflux was higher in dogs receiving butorphanol; however, the difference between the methadone- and the butorphanol-treated groups was not statistically significant [26]. Therefore, although butorphanol, as a κ opioid receptor agonist, slightly affects the intestinal smooth muscle activity as compared with μ agonists [27], relaxation of the oesophageal or pyloric sphincter after methadone administration could not be ruled out.

On contrary, studies concerning the effect of epidural anaesthesia on GER are lacking. In humans, epidural anaesthesia administered as an adjunctive to general anaesthesia is associated with less overall impairment of gastric motility and with a lower gastrin secretion if compared with general anaesthesia alone [28]. In anesthetised children receiving caudal epidural anaesthesia, transient episodes of GER were recorded in 2 out of 16 patients [29]. In detail, in this study, a child developed GER soon after the performance of the local anaesthetic technique. The authors hypothesised that this finding was produced by the changing position of the patient from lateral into dorsal recumbency after the epidural injection [29]. Similarly, in the present study, dogs in the ORT group were first positioned in sternal recumbency for intubation, pH-probe insertion, and epidural injection and then moved into the position required for the surgical procedure. While all the dogs in the END group were maintained in left lateral recumbency, the dogs in the ORT group were placed in dorsal or lateral recumbency, depending on the type of procedure to be performed. It is unlikely that this difference influenced the results obtained in the present cohort. In fact, in a previous study, the authors found no significant differences in the incidence of GER in dogs placed in sternal, dorsal, left lateral, or right lateral recumbency [2]. However, we cannot exclude that the changing position contributed to the pH fluctuations recorded.

In the present study, the mean first pH value did not differ significantly between the two groups and was 6.8 ± 2.1 in the ORT group and 8.2 ± 1.5 in the END group. This finding may suggest the presence of biliary reflux in the END group shortly after the induction of general anaesthesia and before the beginning of the endoscopic procedure. In anaesthetised dogs, reflux is more likely to occur briefly after the induction of anaesthesia [2,3,30]. When GER occurs during or soon after induction, it can be associated with the risk of aspiration pneumonia [30]. However, in our cohort, the dogs did not develop any respiratory complication or any sign of pneumonia at the recovery or the following days.

The measurement of oesophageal pH, by introducing an oesophageal probe close to the lower oesophageal sphincter, was considered the gold standard for the diagnosis of GER in human and in veterinary medicine for many years [8,31]. In fact, the pH monitoring had been shown to have a higher sensitivity, specificity, and accuracy (up to 96%) when compared with manometric examinations of the sphincter tone or direct endoscopic visualisation [8,31]. The main limit of the pH monitoring consists in the inability to detect less acidic or non-acidic episodes [4,32]. Since its introduction in the 1990s, intraluminal impedance resulted as being suitable for monitoring substances moving in the gastrointestinal tract around the probe [32,33]. Therefore, intraluminal impedance has been considered an effective tool for detecting the presence and the extent of GER [32]. Nowadays, combined impedance–pH monitoring is considered the gold standard for diagnosis of GER-related disease [34]. The limit of the present study is that only pH monitoring was performed and therefore the lack of a intraluminal impedance evaluation might have resulted in an underestimation of frequency of episodes of GER, as previously observed in anaesthetised dogs and cats [35,36]. However, the equipment for pH monitoring is cheaper and more readily available in the clinical practice if compared with the impedance technique.

The overall incidence of GER was 87.5%, which is higher than that previously reported in anesthetised dogs, even if different anaesthetic protocols were used [1,2,3,4]. In their study regarding dogs undergoing endoscopy after premedication with butorphanol or methadone, McFadzean and colleagues [26] reported an overall incidence of oesophageal and duodenal reflux of 15% and 45%, respectively. On the contrary, in the present study, a higher incidence of acidic or biliary reflux was observed. This difference was likely related to the technique used for detecting GER; in this study, a pH meter was used, whereas McFadzean and colleagues evaluated the presence of reflux by means of direct endoscopic evaluation. However, McFadzean and colleagues also hypothesised an additive effect of acepromazine and opioids in determining GER [26]. Therefore, irrespective of the procedure to be performed, acidic or biliary reflux could be an expected complication in acepromazine- and opioid-sedated dogs. In veterinary practice, acepromazine is commonly used in combination with methadone or butorphanol for the premedication of canine patients [37]. Additional studies are needed to evaluate the risk of developing GER in dogs sedated with other anaesthetic protocols commonly used in clinical practice, as well as additional studies including a larger number of dogs.

## 5. Conclusions

In conclusion, on the basis of the results of pH meter evaluation, in dogs undergoing general anaesthesia for endoscopic evaluation as well as for non-abdominal surgical procedure, both acidic and biliary GER can be an expected complication, even without any clinical signs of GER (regurgitation and flow of gastric content from the mouth). Therefore, at the end of an endoscopy, attention should be paid to examining the oesophagus directly by means of endoscopic evaluation to rule out episodes of silent GER.

## Figures and Tables

**Table 1:** Demographic data of dogs included in the study undergoing orthopaedic surgery (ORT) or endoscopic examination (END). Data are reported as mean ± standard deviation (SD). * Statistically significant difference.

Parameter	ORT	END
Dogs (*n*)	12	12
Age (months)	70.7 ± 45.1	83.4 ± 45.1
Weight (kg)	24.7 ± 11.5	25.6 ± 9.4
Gender (*n*)	6 males/6 females	8 males/4 females
Duration of anaesthesia (min)	133.3 ± 34.6 *	31.3 ± 9.9 *
Propofol (mg/kg)	2.4 ± 0.9	2.3 ± 0.6

**Table 2:** Episodes of gastroesophageal reflux and details of the pH measurements in dogs undergoing orthopaedic surgery (ORT) or endoscopic examination (END). Data are reported as mean ± standard deviation (SD). * Statistically significant difference (*p* < 0.05).

Parameters	ORT	END	*p* Value
Animals with GER (*n*)	10 (6B/4A)	11 (9B/4A)	0.67 (B)/0.68 (A)
First value (pH)	6.8 ± 2.1	8.2 ± 1.5	0.23
Minimum value (pH)	5.3 ± 2.8	4.6 ± 3.3	0.6
Maximum value (pH)	7.5 ± 2.5	8.6 ± 1.4	0.23
Maximum difference versus first value	−0.7 ± 2.5 *	−2.6 ± 3.5 *	0.25

## References

[1] Galatos A.D., Raptopoulos D. (1995). Gastro-oesophageal reflux during anaesthesia in the dog, the effect of preoperative fasting and premedication. Vet. Rec..

[2] Galatos A.D., Raptopoulos D. (1995). Gastro-oesophageal reflux during anaesthesia in the dog, the effect of age; positioning and type of surgical procedure. Vet. Rec..

[3] Wilson D.V., Evans A.T., Miller R. (2005). Effects of preanesthetic administration of morphine on gastroesophageal reflux and regurgitation during anesthesia in dogs. Am. J. Vet. Res..

[4] Favarato E.S., de Souza M.V., Costa P.R., Pompermayer L.G., Favarato L.S., Ribeiro Júnior J.I. (2011). Ambulatory esophageal pHmetry in healthy dogs with and without the influence of general anesthesia. Vet. Res. Commun..

[5] Adamama-Moraitou K.K., Rallis T.S., Prassinos N.N., Galatos A.D. (2002). Benign oesophageal stricture in the dog and cat: A retrospective study of 20 cases. Can. J. Vet. Res..

[6] Sellon R.K., Willard M.D. (2003). Esophagitis and esophageal strictures. Vet. Clin. N. Am. Small Anim. Pract..

[7] Wilson D.V., Walshaw R. (2004). Postanesthetic esophageal dysfunction in 13 dogs. J. Am. Anim. Hosp. Assoc..

[8] Gawad K.A., Wachowiak R., Rempf C., Tiefenbacher W.J., Strate T., Achilles E.G., Blöchle C., Izbicki J.R. (2003). Ambulatory long-term pH monitoring in pigs. Surg. Endosc..

[9] Savvas I., Rallis T., Raptopoulos D. (2009). The effect of pre-anaesthetic fasting time and type of food on gastric content volume and acidity in dogs. Vet. Anaesth. Analg..

[10] Anagnostou T.L., Savvas I., Kazakos G.M., Ververidis H.N., Psalla D., Kostakis C., Skepastianos P., Raptopoulos D. (2015). The effect of the stage of the ovarian cycle (anoestrus or dioestrus) and of pregnancy on the incidence of gastro-oesophageal reflux in dogs undergoing ovariohysterectomy. Vet. Anaesth. Analg..

[11] Zoran D.L. (2001). Gastroduodenoscopy in the Dog and Cat. Vet. Clin. N. Am. Small Anim. Pract..

[12] Simpson K.W., Jergens A.E. (2011). Pitfalls and progress in the diagnosis and management of canine inflammatory bowel disease. Vet. Clin. N. Am. Small Anim. Pract..

[13] Slovak J., Wang C., Morrison J., Deitz K., LeVine D., Otoni C., King R., Gerber L., Hanson K., Lundberg A. (2014). Endoscopic Assessment of the Duodenum in Dogs with Inflammatory Bowel Disease. J. Vet. Int. Med..

[14] Tams T.R., Tams T.R., Rawling C.A. (2011). Gastroscopy. Small Animal Endoscopy.

[15] Sherding R.G., Johnson S.E., Tams T.R., Rawling C.A. (2011). Esophagoscopy. Small Animal Endoscopy.

[16] Waterman A.E., Hashim M.A. (1991). Measurement of the length and position of the lower oesophageal sphincter by correlation of external measurements and radiographic estimations in dogs. Vet. Rec..

[17] Pietra M., Gianella P. (2018). Bowel endoscopic examination in chronic enteropathy of dog and cat. Veterinaria (Cremona).

[18] Nimmo W.S. (1984). Effect of anaesthesia on gastric motility and emptying. Br. J. Anaesth..

[19] Hardy J.F. (1988). Large volume gastroesophageal reflux, a rationale for risk reduction in the perioperative period. Can. J. Anaesth..

[20] Strombeck D.R., Harrold D. (1985). Effects of atropine; acepromazine; meperidine; and xylazine on gastroesophageal sphincter pressure in the dog. Am. J. Vet. Res..

[21] Hall J.A., Magne M.L., Twedt D.C. (1987). Effect of acepromazine; diazepam; fentanyl-droperidol; and oxymorphone on gastroesophageal sphincter pressure in healthy dogs. Am. J. Vet. Res..

[22] Sternini C., Patierno S., Selmer I.S., Kirchgessner A. (2004). The opioid system in the gastrointestinal tract. Neurogastroenterol. Motil..

[23] Rattan S., Goyal R.K. (1983). Identification and localization of opioid receptors in the opossum lower esophageal sphincter. J. Pharmacol. Exp. Ther..

[24] González E.S., Bellver V.O., Jaime F.C., Cortés J.A., Gil V.G. (2015). Opioid-induced lower esophageal sphincter dysfunction. J. Neurogastroenterol. Motil..

[25] Dowlatshahi K., Evander A., Walther B., Skinner D.B. (1985). Influence of morphine on the distal oesophagus and the lower oesophageal sphincter—A manometric study. Gut.

[26] McFadzean W.J., Hall E.J., van Oostrom H. (2017). Effect of premedication with butorphanol or methadone on ease of endoscopic duodenal intubation in dogs. Vet. Anaesth. Analg..

[27] World Health Organisation (2006). Critical review of butorphanol. Dissertation 34th Meeting of the Expert Committee on Drug Dependence 4.1.

[28] Lombardo L., Ruggia O., Crocellà L., Masoero G., Foti M., Mambrini S., Palombo D., Melchiorri C., Lupo M., Pera A. (2009). Epidural plus general anesthesia vs general anesthesia alone for elective aortic surgery: Effects on gastric electrical activity and serum gastrin secretion. Minerva Anestesiol..

[29] Jean G., Cortambert F., Roy P., Foussat C., Moussa M., Dodat H., Bertrix L. (1992). Reflux gastrooesophagien sous anesthésie caudale et halothane au masque chez l’enfant [Gastroesophageal reflux with combined caudal and halothane anesthesia in children]. Ann. Fr. Anesth. Reanim..

[30] Flouraki E., Savvas I., Kazakos G., Anagnostou T., Bourgazli A. (2020). Aspiration of gastric contents following a gastro-oesophageal reflux episode during general anaesthesia in a dog. Hell. J. Comp. Anim. Med..

[31] Fuchs K.H., DeMeester T.R., Albertucci M. (1987). Specificity and sensitivity of objective diagnosis of gastroesophageal reflux disease. Surgery.

[32] Bredenoord A.J. (2008). Impedance-pH monitoring: New standard for measuring gastro-oesophageal reflux. Neurogastroenterol. Motil..

[33] Silny J. (1991). Intraluminal Multiple Electric Impedance Procedure for Measurement of Gastrointestinal Motility. Neurogastroenterol. Motil..

[34] Frazzoni M., de Bortoli N., Frazzoni L., Tolone S., Savarino V., Savarino E. (2017). Impedance-ph monitoring for diagnosis of reflux disease: New perspectives. Dig Dis. Sci..

[35] Zacuto A.C., Marks S.L., Osborn J., Douthitt K.L., Hollingshead K.L., Hayashi K., Kapatkin A.S., Pypendop B.H., Belafsky P.C. (2012). The influence of esomeprazole and cisapride on gastroesophageal reflux during anesthesia in dogs. J. Vet. Int. Med..

[36] Garcia R.S., Belafsky P.C., Della Maggiore A., Osborn J.M., Pypendop B.H., Pierce T., Walker V.J., Fulton A., Marks S.L. (2017). Prevalence of Gastroesophageal Reflux in Cats During Anesthesia and Effect of Omeprazole on Gastric pH. J. Vet. Intern. Med..

[37] Monteiro E.R., Junior A.R., Assis H.M., Campagnol D., Quitzan J.G. (2009). Comparative study on the sedative effects of morphine, methadone, butorphanol or tramadol, in combination with acepromazine, in dogs. Vet. Anaesth. Analg..

